# The AhR pathway is dysregulated in alopecia areata^☆^

**DOI:** 10.1016/j.jtauto.2025.100282

**Published:** 2025-03-07

**Authors:** Arno Belpaire, Annelies Demeyer, Elise Van Caelenberg, Nanja van Geel, Reinhart Speeckaert

**Affiliations:** a Department of Dermatology, Ghent University Hospital, Corneel Heymanslaan 10, 9000, Ghent, Belgium; b Department of Head and Skin, Ghent University, Faculty of Medicine and Health Sciences, Corneel Heymanslaan 10, 9000, Ghent, Belgium

**Keywords:** Alopecia areata, Aryl hydrocarbon receptor, Immune checkpoints, IFN-γ, T cells, Cytotoxicity, Inflammatory skin diseases

## Abstract

Despite significant progress in the treatment of alopecia areata (AA), many aspects of its immune-based pathogenesis remain unexplored. IFN-γ, primarily produced by CD8^+^ T cells and NK cells, is considered central to AA pathogenesis. However, the complex immune signaling network contributes to therapeutic resistance and frequent disease flares after treatment discontinuation. The aryl hydrocarbon receptor (AhR) pathway, upregulated by IFN-γ, modulates Th17 responses, but its inhibitory effects on IFN-γ remain unclear. Although IL-17 levels are elevated in AA, clinical trials indicate that IL-17A inhibitors are ineffective. AhR expression is known to induce immune checkpoints (ICPs) such as PD-1, suggesting a potential role as a negative feedback mechanism. This study investigated AhR expression in lymphocytes from AA patients and its association with clinical and laboratory markers of disease activity. AhR expression was significantly reduced in CD4, CD8, Th1, and Th17 lymphocytes in AA patients compared to healthy controls (*p* < 0.005), and it correlated inversely with SALTII scores (*p* < 0.05). ROC analysis showed that AhR levels in CD8 cells could differentiate mild AA from healthy controls with a sensitivity of 82.35 % and specificity of 86.84 %, suggesting potential diagnostic utility. Lower AhR levels were associated with increased IFN-γ+ lymphocytes and decreased IL-17+ immune cells. Interestingly, immune profiles differed between atopic and non-atopic patients: in severe AA, higher AhR expression was linked to increased sPD-1 concentrations, whereas in limited AA, AhR failed to upregulate any investigated ICP. These findings highlight the significant downregulation of the AhR pathway in AA and suggest its potential as a therapeutic target. Future research should explore the development of AhR agonists or antagonists to modulate immune responses in AA.

## Abbreviations

**AA**: Alopecia Areata
**AhR**: Aryl Hydrocarbon Receptor
**AUC**: Area Under the Curve
**BMI**: Body Mass Index
**BTLA**: B- and T-Lymphocyte Attenuator
**CCR4**: C-C Chemokine Receptor Type
**CCR6**: C-C Chemokine Receptor Type 6
**CD**: Cluster of Differentiation
**CCL13/MCP-4**: Chemokine (C-C motif) Ligand 13/Monocyte Chemotactic Protein 4
**CCL18/PARC**: Chemokine (C-C motif) Ligand 18/Pulmonary and Activation-Regulated Chemokine
**CCL24/Eotaxin-2**: Chemokine (C-C motif) Ligand 24/Eotaxin-2
**CCL26/Eotaxin-3**: Chemokine (C-C motif) Ligand 26/Eotaxin-3
**CTLA-4**: Cytotoxic T-Lymphocyte-Associated Protein 4
**FICZ**: 6-Formylindolo[3,2-b]carbazole
**FMO**: Fluorescence Minus One
**FSC**: Forward Scatter
**GM-CSF**: Granulocyte-Macrophage Colony-Stimulating Factor
**HVEM**: Herpesvirus Entry Mediator
**ICP**: Immune Checkpoint Protein
**IDO**: Indoleamine 2,3-Dioxygenase
**IFN-γ**: Interferon-Gamma
**IgE**: Immunoglobulin E
**IL**: Interleukin
**JAK**: Janus Kinase
**JAK-STAT Pathway**: Janus Kinase-Signal Transducer and Activator of Transcription Pathway
**KynA**: Kynurenic Acid
**MFI**: Mean Fluorescence Intensity
**MHC**: Major Histocompatibility Complex
**NKG2D**: Natural Killer Group 2D Receptor
**NK Cells**: Natural Killer Cells
**PBMCs**: Peripheral Blood Mononuclear Cells
**PD-1**: Programmed Death-1
**PD-L1/PD-L2**: Programmed Death-Ligands 1 and 2
**ROC**: Receiver Operating Characteristic
**SALT II**: Severity of Alopecia Tool II
**sPD-1**: Soluble Programmed Death-1
**SSC**: Side Scatter
**TCDD**: 2,3,7,8-Tetrachlorodibenzo-p-dioxin
**TDO**: Tryptophan 2,3-Dioxygenase
**TGF-β**: Transforming Growth Factor-Beta
**Th Cells**: T Helper Cells
**TIM-3**: T-Cell Immunoglobulin and Mucin-Domain Containing-3
**TNF**: Tumor Necrosis Factor
**Tr1 Cells**: Type 1 Regulatory T Cells
**Tregs**: Regulatory T Cells
**Trp**: Tryptophan

## 1 Introduction

Alopecia areata (AA) is a common inflammatory skin disease characterized by the loss of hair, typically in patches on the scalp [1]. The pathophysiology of AA has garnered increasing interest due to the emergence of successful new therapeutics, particularly janus kinase (JAK) inhibitors [2]. Central to the immune-mediated hair loss in AA is the cytokine interferon-gamma (IFN-γ) [3]. Mice lacking the IFN-γ gene exhibit resistance to developing AA [4]. Additionally, studies have shown that serum IFN-γ levels are elevated in individuals with AA, and these levels correlate with both the duration and extent of the disease, reaching their peak in the most severe forms, alopecia totalis and universalis [5].

A critical aspect of AA pathogenesis is the disruption of "immune privilege" within hair follicles. Immune privilege refers to the phenomenon where certain tissues, such as hair follicles, are shielded from immune surveillance and attack. This protection is maintained by various mechanisms, including reduced expression of major histocompatibility complex (MHC) class I and II molecules, the presence of immunosuppressive factors, and the activity of regulatory immune cells. In AA, this immune privilege is compromised, leading to an influx of immune cells, primarily NKG2D + T cells and natural killer (NK) cells, into the hair follicles. These cells, driven by IFN-γ and potentially other factors, initiate an inflammatory response that targets the hair follicles, resulting in hair loss [6]. The loss of immune privilege in AA may be attributed, in part, to decreased activity of immune checkpoint proteins (ICPs). ICPs, including the programmed death (PD)-1/PD-L1 pathway, contribute to the maintenance of immune privilege in hair follicles [6]. Notably, AA has been observed as an immune-related adverse event in patients receiving ICP inhibitors, which is associated with a favorable response in cancer treatment [7]. Unfortunately, little information is available on the expression of immune checkpoints in AA, highlighting an area for further research.

While the crucial role of IFN-γ is well-established, the involvement of other cytokines in AA pathogenesis is more intricate. Research has indicated an increase in various cytokines in AA, including IL-2, IL-5, IL-6, TNF, IL-12, IL-17, IL-17E, and IL-22, some of which demonstrate correlations with disease severity and duration [8,9]. Conversely, the anti-inflammatory cytokine IL-35, known to support the development of regulatory T cells (Tregs), is generally found to be decreased in AA [10]. The effectiveness of JAK inhibitors in treating AA can be attributed to their capacity to inhibit IFN-γ signaling. These inhibitors primarily target JAK1 and JAK2, which are involved in IFN-γ signal transduction. While JAK3 inhibition has also demonstrated some benefit in AA, its primary role is in the signaling of other cytokines [11]. Despite the progress JAK inhibitors represent, long-term treatment is frequently necessary in most cases, and the risk of relapse remains significant, even with a gradual tapering of the medication. Furthermore, concerns regarding the long-term safety of JAK inhibitors persist, particularly in older patients with comorbidities [12]. Although an impressive step forward, the search for other targeted treatments for alopecia areata continues.

The observation of increased IL-17 levels in AA initially suggested that targeting this cytokine could be a promising therapeutic approach. However, a pilot study conducted at our department using the IL-17 inhibitor secukinumab did not demonstrate clinical efficacy. Furthermore, reports have documented the onset of AA in patients undergoing treatment with anti-IL-17 biologics [13,14]. These findings suggest that the upregulation of the Th17 pathway, which involves IL-17, may not be a primary driver of hair loss in AA.

Atopic diseases—such as atopic dermatitis, asthma, and allergic rhinitis—are common comorbidities in AA and define an immunologically distinct subgroup. The anti-IL-4R biologic dupilumab has proven effective in stimulating hair regrowth in atopic AA patients, particularly those with elevated IgE levels, while showing significantly less efficacy in non-atopic patients. This suggests that IgE measurement could be a valuable biomarker for predicting a positive response to dupilumab. Immunological studies, including a randomized clinical trial, have demonstrated that dupilumab treatment leads to downregulation of the Th2 signature—marked by reductions in Th2-related markers such as CCL13/MCP-4, CCL18/PARC, CCL26/eotaxin-3, and CCL24/eotaxin-2—and subsequent suppression of Th1 signaling. Patients with a history of atopy or elevated baseline serum IgE levels experienced significantly greater hair regrowth compared to those without atopy and low IgE levels, underscoring the importance of identifying atopic AA patients for dupilumab treatment [15]. The mechanism of action of dupilumab might be explained by the intricate interplay between cytokines. IL-4 and TGF-β, both known to inhibit IFN-γ, exhibit mutual inhibition when their levels are both elevated, effectively negating their suppressive effect on IFN-γ. Blocking IL-4 with dupilumab may restore the inhibitory action of TGF-β on IFN-γ, highlighting the complex pathogenesis of AA [16].

Given the limitations of current treatments, exploring alternative pathways involved in AA pathogenesis is crucial. The AhR pathway has not yet been investigated in depth in AA. AhR activity prevents excessive inflammation in tissues and barrier organs such as the skin, gut, and lungs. Its signaling cascade is highly complex, involving both endogenous and exogenous ligands, highlighting the influence of environmental triggers such as diet and microbiota on the development of autoimmune diseases [17]. Kynurenine, an endogenous ligand and a degradation product of tryptophan, activates AhR. The metabolism of tryptophan is regulated by the enzymes indoleamine 2,3-dioxygenase (IDO) and tryptophan 2,3-dioxygenase (TDO) [18]. IDO is strongly upregulated following IFN-γ exposure and, due to its immune-dampening effects, is considered an important ICP in cytotoxic immune responses [19]. The AhR pathway is also involved in the upregulation of other immune checkpoints such as PD-1 [20]. Additionally, AhR mediates the upregulation of PD-L1 and IDO by IFN-γ via the JAK-STAT pathway [21]. This suggests that AhR could be an important factor in the negative feedback mechanism in IFN-γ–mediated autoimmune disorders.

In this study, we analyzed AhR expression in CD4^+^, Th1, Th17, and CD8^+^ lymphocytes in patients with AA. We further investigated the associations between AhR expression and the balance of IFN-γ and IL-17–producing lymphocytes. Given the link between AhR and both IDO and PD-L1, we also explored correlations between AhR expression and a broad range of ICPs. This observational approach provides preliminary insights into the potential of the AhR pathway as a future therapeutic target in AA.

## 2 Methods

### 2.1 Patient recruitment and sample collection

Patients diagnosed with AA were consecutively enrolled at the Department of Dermatology, Ghent University Hospital. Healthy control subjects without a history of autoimmune diseases were also recruited for comparison. The study received approval from the Ethics Committee of Ghent University Hospital, and written informed consent was obtained from all participants prior to inclusion.

Clinical data collected included age, sex, age of onset, disease duration, body mass index (BMI), smoking status, family history of autoimmune diseases, and atopic background (clinical history of atopic dermatitis, and/or a history of allergic rhinitis, and/or a history of asthma, in accordance with standard clinical definitions [22]). The severity of AA was assessed using the Severity of Alopecia Tool II (SALT II) score [23]. Peripheral blood samples were collected from all participants. Serum samples were stored at −80 °C, and peripheral blood mononuclear cells (PBMCs) were isolated and preserved in liquid nitrogen until further analysis.

### 2.2 Flow cytometry

Flow cytometry was performed to evaluate the expression of AhR and cytokine production in various T cell subsets. PBMCs were thawed and stained with fluorescently labeled antibodies targeting specific cell surface markers and intracellular proteins. Th1 cells were characterized as CD3^+^CD4^+^CD161^−^CCR6^-^ cells, and Th17 cells as CD3^+^CD4^+^CD161^+^CCR6^+^CCR4^+^ cells, following established gating strategies [24].

Non-lymphocytes were gated sequentially: FSC/SSC gating to include all cells, singlet gating, live/dead viability gating, and exclusion of CD3^+^ cells to isolate CD3^−^ populations. The final non-lymphocyte gate was applied on an FSC/SSC plot. For lymphocyte subsets, FSC/SSC gating isolated lymphocytes, followed by singlet, live/dead, and CD3^+^ gating to identify T cells. CD4^+^ and CD8^+^ subsets were distinguished through subsequent gating. Data quality was ensured at the end using the PeacoQC plugin.

The mean fluorescence intensity (MFI) of AhR was measured in CD4^+^ T cells, CD8^+^ T cells, Th1 cells, Th17 cells and non-lymphocytes. To determine the net MFI of AhR expression, the MFI value of the fluorescence minus one (FMO) control was subtracted from the MFI of the target population. Intracellular cytokine staining was conducted to assess the production of interferon-gamma (IFN-γ), interleukin-17 (IL-17), and granulocyte-macrophage colony-stimulating factor (GM-CSF) in the aforementioned lymphocyte subsets.

Flow cytometry data were acquired on a BD FACSFortessa™ X-20 (Becton, Dickinson and Company, Franklin Lakes, NJ, USA) and analyzed with FlowJo™ software version 10 (Becton, Dickinson and Company, Franklin Lakes, NJ, USA).

### 2.3 Multiplex bead-based immunoassay

To explore the relationship between AhR expression, immune activation markers, and ICPs, serum levels of selected proteins were measured using a multiplex bead-based immunoassay. The ProcartaPlex™ Human Immuno-Oncology Checkpoint Panel 1 (Thermo Fisher Scientific, Waltham, MA, USA) was utilized to quantify circulating levels of immune checkpoints and activation markers, including PD-1, PD-L1, PD-L2, cytotoxic T-lymphocyte-associated protein 4 (CTLA-4), lymphocyte activation gene 3 (LAG-3), T-cell immunoglobulin, IDO and mucin-domain containing-3 (TIM-3), B- and T-lymphocyte attenuator (BTLA), herpesvirus entry mediator (HVEM), CD27, CD28, CD80, and CD137.

PBMCs were also stimulated using Dynabeads® Human T-Activator CD3/CD28 (Thermo Fisher Scientific) at a cell-to-bead ratio of 1:1 for 22 h to mimic T-cell activation. Supernatants were collected post-stimulation and analyzed using the same multiplex panel to assess the secretion of immune checkpoints and activation markers upon cellular activation.

### 2.4 Statistical analysis

Data analysis was performed using IBM SPSS Statistics version 30.0.0 (IBM Corp., Armonk, NY, USA), JASP version 0.19.1 (JASP Team, Amsterdam, Netherlands), and MedCalc Statistical Software version 22.032 (MedCalc Software Ltd., Ostend, Belgium). The Shapiro-Wilk test was employed to assess the normality of continuous variables. For non-parametric data, the Mann-Whitney *U* test was used for comparisons between two groups, and the Kruskal-Wallis test was applied when comparing more than two groups.

Correlation analyses were conducted using Pearson's correlation coefficient for normally distributed data and Spearman's rank correlation coefficient for non-parametric data. Heat maps were generated using JASP to visualize correlation patterns. In analyses comparing atopic and non-atopic patients, partial correlation was performed to control for the SALT II score, minimizing bias due to differences in disease severity.

Receiver Operating Characteristic (ROC) curve analysis was utilized to evaluate the diagnostic potential of AhR expression levels in distinguishing between limited (SALT II ≤ 25 %) and extensive (SALT II > 25 %) AA, as well as between AA patients and healthy controls. The optimal cutoff values were determined using the Youden index, with corresponding sensitivity and specificity calculated.

Multiple linear regression models were employed to identify independent predictors of AhR expression and cytokine production. A significance level of *p* < 0.05 was set for all statistical tests.

## 3 Results

### 3.1 Patient characteristics

A total of 36 patients with AA and 38 healthy controls were included in this study. Among the AA patients, 23 (63.9 %) were women, with a mean age of 43.2 years (median: 47.5 years; IQR: 32–51.3 years). The patients had a relatively long disease duration, with a mean of 12.2 years (median: 8 years; IQR: 4–18.5 years). The SALTII score was 43.3 % (median: 30.5 %; IQR: 9–91.5 %). Based on patient history and clinical examination, only 2 patients with patchy AA were identified as being in a chronic phase (5.6 %), while 22 presented in an acute phase (61.1 %), 8 had alopecia totalis/universalis (22.2 %), and 4 were unclear (11.1 %).

At the time of blood sampling, five patients (13.9 %) were receiving systemic treatment, while the remaining patients were either untreated or managed with topical or intralesional corticosteroids. Specifically, one patient was on a JAK inhibitor, one on dupilumab, one on cyclosporin, and two on methotrexate. Given that these therapies may modulate immune pathways in distinct ways, we conducted a sensitivity analysis to assess their potential impact on our findings. While we included these patients in our primary analysis, we performed an additional analysis excluding them to determine whether their presence skewed our results. Importantly, the findings for AhR expression remained entirely consistent, and statistical significance was retained. Therefore, we concluded that their inclusion did not bias our results and opted to maintain them in our primary dataset. Additionally, 16 patients (44.4 %) had an atopic background, including a history of atopic dermatitis, asthma, or allergic rhinitis.

### 3.2 AhR expression in lymphocytes in alopecia areata

The MFI of AhR was highest in Th17 cells (AhR Th17 vs. AhR CD4^+^ T cells: *p* < 0.001), followed by CD8^+^ T cells (AhR CD8^+^ vs. AhR CD4^+^ T cells: *p* = 0.034), Th1 cells, and the overall group of CD4^+^ Th cells (Fig. 1A). The MFI of AhR was significantly higher in healthy controls compared to patients with AA across all investigated T cell subsets, including CD4^+^ T cells (*p* = 0.002), Th1 cells (*p* = 0.003), Th17 cells (*p* < 0.001), and CD8^+^ T cells (*p* < 0.001).

**Fig. 1:**
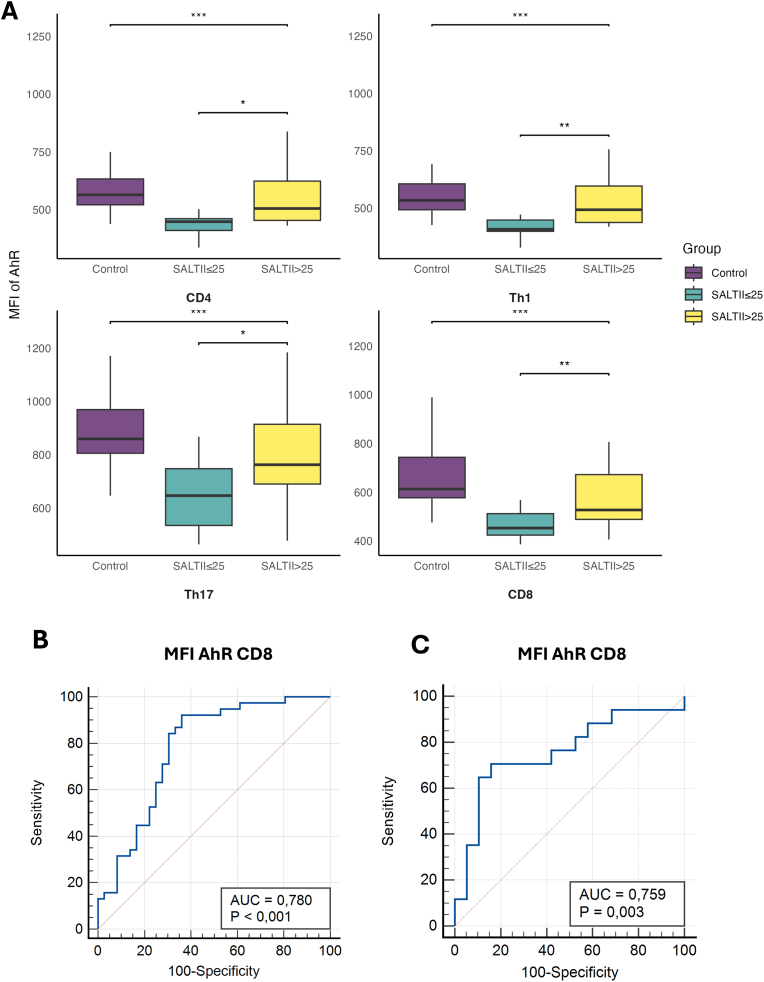
AhR expression and diagnostic potential in AA. (A) Boxplot showing the MFI of AhR in CD4, Th1, Th17, and CD8 lymphocytes across three groups: healthy controls, AA patients with limited disease extent (SALTII ≤25 %) and AA patients with extensive disease extent (SALTII >25 %). AhR expression was significantly higher in healthy controls compared to AA patients across all lymphocyte subsets. Among AA patients, those with SALTII ≤25 % had lower AhR expression compared to patients with SALTII >25 %, indicating a potential link between disease severity and AhR expression levels. (B) ROC curve analysis of the MFI of AhR in CD8 lymphocytes to distinguish AA patients from healthy controls. An AUC of 0.780 was achieved, with a sensitivity of 82.35 % and specificity of 86.84 %. (C) ROC curve analysis of the MFI of AhR in CD8 lymphocytes to predict extensive AA (SALTII >25 %) compared to limited AA (SALTII ≤25 %). The analysis demonstrated an AUC of 0.759, with a sensitivity of 70.59 % and specificity of 84.21 %, suggesting the diagnostic potential of AhR expression as a biomarker for disease extent in AA. **Abbreviations:** MFI, mean fluorescence intensity; AhR, aryl hydrocarbon receptor; ROC, Receiver Operating Characteristic; AUC, area under the curve; AA, alopecia areata; SALTII, Severity of Alopecia Tool II.

Interestingly, AhR expression was lower in patients with a SALT II score ≤25 % compared to those with a SALT II score >25 %. This difference was significant for all examined T cell subtypes: CD4^+^ T cells (*p* = 0.011), Th1 cells (*p* = 0.007), Th17 cells (*p* = 0.010), and CD8^+^ T cells (*p* = 0.008). When comparing healthy controls to alopecia areata patients with a SALT II score >25 %, no significant differences in AhR expression were observed, except for higher AhR expression in CD8^+^ T cells in the patient group (*p* = 0.018).

No significant differences in AhR expression were observed in the non-lymphocyte population when comparing controls, AA patients with SALT II > 25 %, and AA patients with SALT II ≤ 25 % (Supplementary Fig. 2).

Receiver Operating Characteristic (ROC) curve analysis demonstrated that the MFI of AhR in Th17 lymphocytes could discriminate between AA patients and healthy controls, with an area under the curve (AUC) of 0.780 (*p* < 0.001) (Fig. 1B). The optimal cutoff point provided a sensitivity of 92.11 % and a specificity of 63.89 %. ROC analysis for predicting extensive AA (SALT II > 25 % vs. SALT II ≤ 25 %) yielded an AUC of 0.759, with an optimal cutoff resulting in a sensitivity of 70.59 % and specificity of 84.21 % (Fig. 1C). The most significant diagnostic value of AhR expression was observed when comparing healthy controls with patients having limited disease extent (SALT II ≤ 25 %). Specifically, the MFI of AhR in CD8^+^ lymphocytes achieved a sensitivity of 82.35 % and a specificity of 86.84 % (AUC = 0.876; *p* < 0.001).

Correlation analysis revealed that AhR expression in Th17 cells positively correlated with the SALT II score (*r* = 0.355; *p* = 0.034), whereas no such correlation was found for CD4^+^ T cells, Th1 cells, or CD8^+^ T cells (Fig. 2). However, when patients with alopecia universalis (*n* = 8) were excluded from the analysis, a strong significant correlation between disease extent and AhR expression emerged for all T cell subsets examined: CD4^+^ T cells (*r* = 0.458; *p* = 0.014), Th1 cells (*r* = 0.453; *p* = 0.016), Th17 cells (*r* = 0.518; *p* = 0.005), and CD8^+^ T cells (*r* = 0.543; *p* = 0.003).

**Fig. 2:**
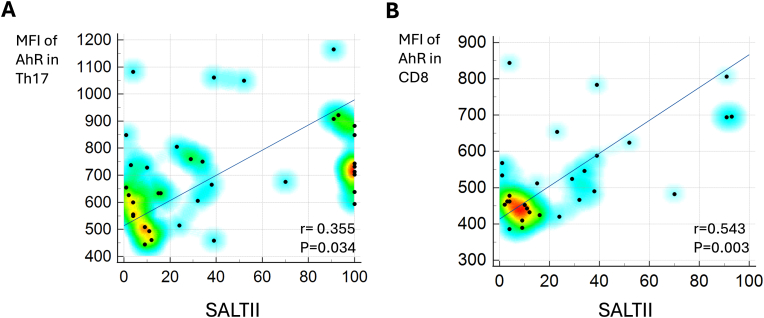
Correlation between AhR expression and disease extent in AA. (A) Pearson's correlation between the MFI of AhR in Th17 lymphocytes and the SALTII score, demonstrating a significant positive relationship. (B) Pearson's correlation between the MFI of AhR in CD8 lymphocytes and the SALTII score after exclusion of patients with alopecia totalis, showing a stronger positive correlation when these cases are excluded. **Abbreviations**: MFI, mean fluorescence intensity; AhR, aryl hydrocarbon receptor; SALTII, Severity of Alopecia Tool II; AA, alopecia areata.

#### 3.2.1 The link between AhR expression and IFN-γ and IL17 producing T cells

**Fig. 3:**
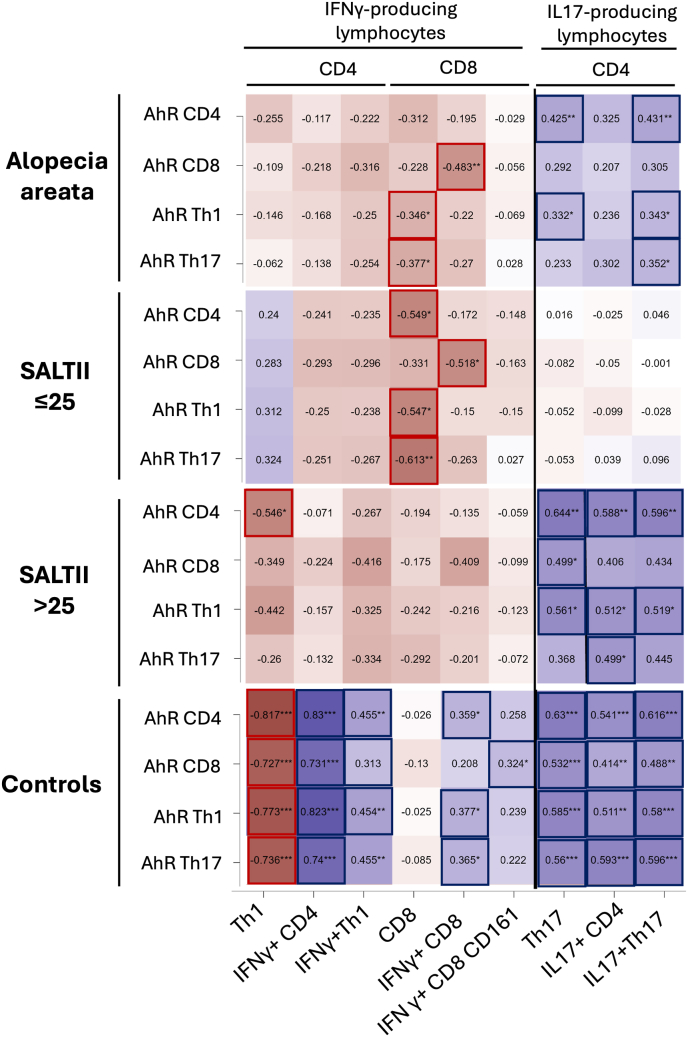
Correlation heatmap between AhR expression and cytokine-producing T lymphocyte subsets in AA. Heatmap illustrating the Pearson's correlation between the MFI of AhR in CD4, CD8, Th1, and Th17 lymphocytes and various IFN-γ and IL-17 producing T lymphocyte subsets, including Th1, IFN-γ^+^CD4, IFN-γ^+^Th1, CD8, IFN-γ^+^CD8, IFN-γ^+^CD8^+^CD161^+^, Th17, IL-17^+^CD4, and IL-17^+^Th17 lymphocytes. Significant correlations are highlighted in red (positive) and blue (negative) boxes. **Abbreviations**: MFI, mean fluorescence intensity; AhR, aryl hydrocarbon receptor; IFN-γ, interferon-gamma; IL-17, interleukin-17; Th1, T helper 1; Th17, T helper 17; AA, alopecia areata.

Overall, a negative correlation was observed between the MFI of AhR on Th cells and IFN-γ–producing CD8^+^ cells in patients with AA (Fig. 3). Specifically, the AhR MFI in Th1 and Th17 lymphocytes was significantly inversely associated with the number of cytotoxic lymphocytes (*r* = −0.346, *p* = 0.039; *r* = −0.388, *p* = 0.020, respectively). Additionally, the AhR MFI in CD8^+^ T cells inversely correlated with the percentage of IFN-γ^+^ CD8^+^ T cells (*r* = −0.483, *p* = 0.003).

Interestingly, the opposite pattern was observed in healthy controls, where positive associations between AhR MFI on Th cells and IFN-γ^+^ CD8^+^ T cells were noted. In these individuals, higher AhR MFI levels were associated with an increased number of interleukin-17 (IL-17)–producing lymphocytes. While a similar trend was seen in patients with extensive AA, this association was entirely absent in patients with a SALT II score ≤25 %. This absence may be due to the low AhR expression in this subgroup, which may not reach the threshold required to induce Th17 activation.

Furthermore, a clear difference between atopic and non-atopic patients was observed (Supplementary Fig. 1). In atopic patients, the correlation between AhR MFI and IFN-γ^+^ CD4^+^ lymphocytes was lacking, although the negative association between AhR MFI levels in CD8^+^ cells and the number of cytotoxic T cells remained significant. Additionally, in atopic AA patients, higher AhR MFI did not lead to increased Th17 responses, in contrast to non-atopic patients. This illustrates the distinct immunological mechanisms leading to AA depending on the presence of underlying atopy.

### 3.3 The link between AhR expression and immune checkpoints in alopecia areata

**Fig. 4:**
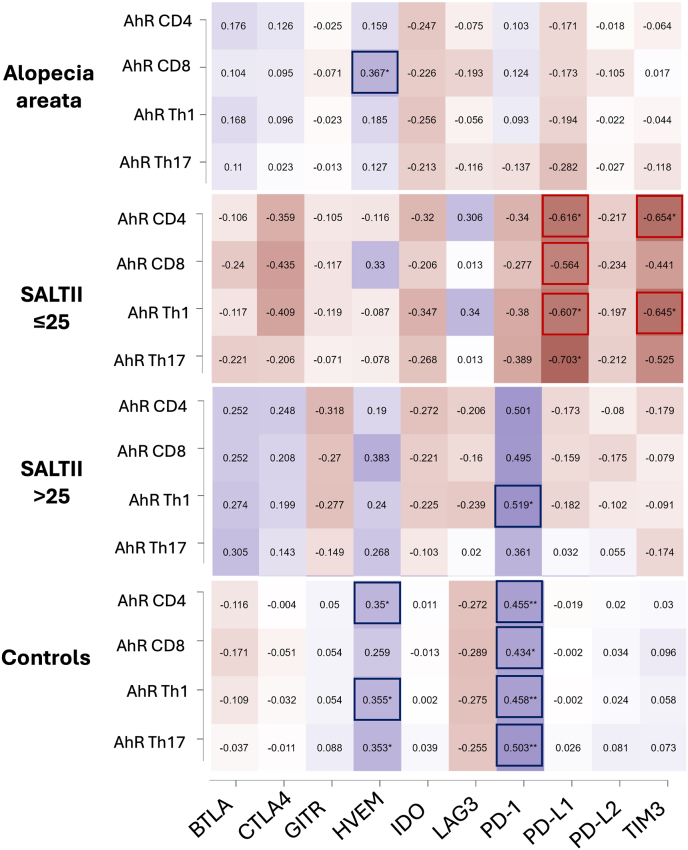
Correlation heatmap between AhR expression and soluble immune checkpoints in AA. Heatmap illustrating the Pearson's correlation between the MFI of AhR in T lymphocyte subsets and soluble immune checkpoints, including BTLA, CTLA-4, GITR, HVEM, IDO, LAG-3, PD-1, PD-L1, PD-L2, and TIM-3. Significant correlations are highlighted in red (positive) and blue (negative) boxes. **Abbreviations**: MFI, mean fluorescence intensity; AhR, aryl hydrocarbon receptor; BTLA, B- and T-lymphocyte attenuator; CTLA-4, cytotoxic T-lymphocyte-associated protein 4; GITR, glucocorticoid-induced tumor necrosis factor receptor-related protein; HVEM, herpesvirus entry mediator; IDO, indoleamine 2,3-dioxygenase; LAG-3, lymphocyte activation gene 3; PD-1, programmed death-1; PD-L1, programmed death-ligand 1; PD-L2, programmed death-ligand 2; TIM-3, T-cell immunoglobulin and mucin-domain containing-3; AA, alopecia areata.

In AA, the MFI of AhR in CD8^+^ T cells positively correlated with HVEM levels in supernatant (*r* = 0.367, *p* = 0.039) (Fig. 4). This finding was similar to that in healthy controls, where a link between AhR expression and increased HVEM levels was also observed. As previously reported, higher AhR expression correlated with elevated levels of sPD-1 in supernatant [25]. This correlation was present in patients with extensive AA (SALT II > 25 %) but was not observed in those with limited disease (SALT II ≤ 25 %).

In patients with limited AA (SALT II ≤ 25 %), AhR expression was negatively correlated with sPD-L1 and sTIM-3. These correlations were not detected in patients with extensive AA or in healthy controls. This difference is likely due to the reduced AhR expression in patients with limited disease, which may not reach the threshold necessary to induce the upregulation of these immune checkpoints.

## 4 Discussion

Our findings demonstrate significantly reduced AhR expression in CD4, CD8, Th1, and Th17 lymphocytes in AA patients compared to healthy controls. This aligns with previous studies reporting decreased AhR expression in other inflammatory skin disorders, including psoriasis, atopic dermatitis, acne, hidradenitis suppurativa, and vitiligo [26].

AhR is a ligand-activated transcription factor highly expressed in the skin and its appendages, such as the interfollicular epidermis, hair follicles, and the inner and outer root sheath regions [27]. Activation of AhR influences transcriptional regulation within the immune system, particularly impacting the differentiation of Th17 and regulatory T cells [28]. Studies in mice have underscored the importance of finely tuning AhR signaling in the skin: complete genetic deletion of *Ahr* results in more severe psoriasis-like inflammation, while constitutively active AhR in keratinocytes causes alterations resembling atopic dermatitis [29]. These findings highlight the critical balance required for proper AhR function in skin immunity.

Interestingly, the observed reduction in AhR expression in AA patients may be primarily restricted to T cells. Our analysis revealed no significant difference in AhR MFI in non-lymphocyte immune cells between AA patients and healthy controls. This observation aligns with previous research demonstrating that AhR expression is Th subset-specific: Th17 cells exhibit the highest levels of AhR, regulatory T cells (Tregs) and Tr1 cells show intermediate expression, while Th0, Th1, and Th2 subsets exhibit minimal AhR expression [17,28]. The observed lower AhR expression in AA patients may be linked to the imbalance of these cell populations.

Despite the overall reduction in AhR expression in AA, our results reveal a nuanced relationship between AhR signaling and disease severity. Patients with SALTII scores >25 exhibited higher AhR expression compared to those with lower SALTII scores, although their AhR levels remained below those of healthy controls. This association may stem from elevated IFN-γ levels in patients with more extensive disease, as IFN-γ induces IDO, which catalyzes the conversion of tryptophan into kynurenine—a known AhR ligand [30]. These findings suggest a complex interplay between AhR signaling, IFN-γ, and disease activity in AA, warranting further investigation to elucidate potential therapeutic implications.

In AA patients with an atopic background, AhR expression was not associated with a reduction in IFN-γ^+^CD4^+^ T cells, in contrast to the findings in non-atopic AA patients. This discrepancy may reflect the distinct T helper cell polarization towards a Th2-dominant response typically observed in atopic AA [15]. Interestingly, AhR expression in CD8^+^ T cells exhibited a consistent negative correlation with the number of CD8 lymphocytes in both atopic and non-atopic patients. This finding suggests that AhR may play a role in regulating CD8 T cell populations in AA, irrespective of the presence of atopy.

Regarding immune checkpoints, soluble PD-1 (sPD-1) in the supernatant of stimulated PBMCs correlated with AhR in patients with extensive AA and healthy controls, while no such correlation was observed in limited AA. In AA with a SALTII ≤25, an inverse correlation with PD-L1 and TIM-3 was noted, suggesting that low AhR in this subgroup may be insufficient to exert a positive influence on negative feedback mechanisms.

AhR ligands could be a promising therapeutic option in AA. The interaction of AhR with transcriptional partners varies depending on the specific ligand. For example, 6-Formylindolo[3,2-b]carbazole (FICZ) promotes the differentiation of Th17 cells, while 2,3,7,8-tetrachlorodibenzo-p-dioxin (TCDD) stimulates regulatory T cell formation, potentially improving autoimmune responses [28]. This suggests that different AhR ligands may have varying efficacy in treating autoimmune disorders.

Keratinocytes express high levels of AhR, even in a resting state, and this expression is further increased during inflammation [31]. Topical administration of L-kynurenine (or a derivative such as KynA) displays anti-fibrotic properties in wound healing and also reduces scarring and lymphocyte-based inflammation [31,32]. Tapinarof, an AhR agonist, is FDA-approved for the topical treatment of psoriasis. In psoriasis, tapinarof reduces the levels of key pathogenic cytokines, including IL-17A, IL-17F, IL-22, and IL-23 [33]. Importantly, IFN-γ is also downregulated following tapinarof treatment in psoriasis and lupus [34,35]. These findings regarding tapinarof's effect on IFN-γ align with the observed link between AhR expression and the downregulation of IFN-γ+ CD4 and CD8 lymphocytes in AA. These findings suggest that modulating AHR activity could potentially suppress the inflammatory cascade driven by IFN-γ and other pro-inflammatory cytokines, offering a new approach to managing AA.

The role of the AhR pathway in hair diseases remains poorly understood. AhR is highly expressed in various compartments of the hair follicle, where it regulates follicular cycling. Its depletion in bulge stem cells disrupts stem cell properties by upregulating differentiation markers [29]. Moreover, aged AhR-deficient mice exhibit dystrophic hair follicles, emphasizing the importance of AhR in maintaining follicular structure and function [36]. In female pattern hair loss, AhR has been reported to be overexpressed in miniaturized follicles, though its precise role—whether contributory or inhibitory—remains unclear [37]. Further research into AhR expression within the hair follicle microenvironment in AA could yield valuable insights.

A limitation in our research was the challenge of differentiating between AhR-positive and AhR-negative groups within the flow cytometry data, leading us to rely on mean fluorescence intensity (MFI) as an alternative measure. The lack of a distinct separation between positive and negative signals suggests that AhR expression varies continuously across a spectrum, rather than displaying a clear bimodal pattern. This continuous variation is also reflected in the data provided on the antibody testing data sheet.

In conclusion, this study demonstrates the decreased expression of AhR in different T lymphocyte subsets and the correlation of AhR with the SALTII score. The inverse link between AhR and IFN-γ producing T lymphocytes points to a pathogenic role. For these reasons, AhR ligands might be an attractive treatment option that can be further explored.

## CRediT authorship contribution statement

**Arno Belpaire:** Writing – review & editing, Writing – original draft, Visualization, Software, Resources, Project administration, Methodology, Investigation, Funding acquisition, Formal analysis, Data curation, Conceptualization. **Annelies Demeyer:** Writing – review & editing, Supervision, Methodology. **Elise Van Caelenberg:** Writing – review & editing. **Nanja van Geel:** Writing – review & editing. **Reinhart Speeckaert:** Writing – review & editing, Visualization, Validation, Supervision, Project administration, Methodology, Funding acquisition, Formal analysis, Conceptualization.

## Declaration of generative AI and AI-assisted technologies in the writing process

During the preparation of this work the author(s) used ChatGPT in order to improve language and readability. After using this tool/service, the author(s) reviewed and edited the content as needed and take(s) full responsibility for the content of the publication.

## Funding

This research was funded by the Research Foundation - Flanders (10.13039/501100003130FWO), Aspirant Fundamental Research, grant number 1125825N.

## Declaration of competing interest

The authors declare that they have no known competing financial interests or personal relationships that could have appeared to influence the work reported in this paper.

## Figures and Tables

**figs1:**
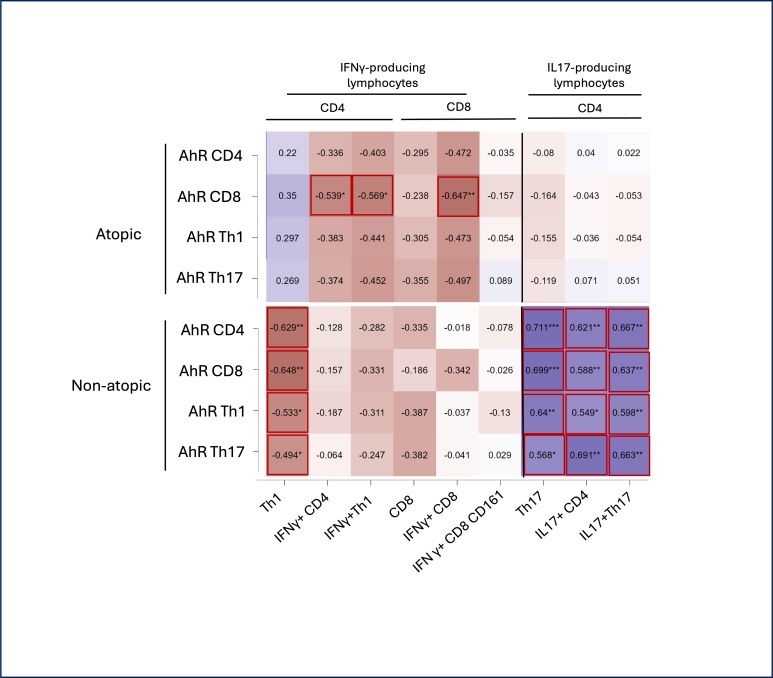


**figs2:**
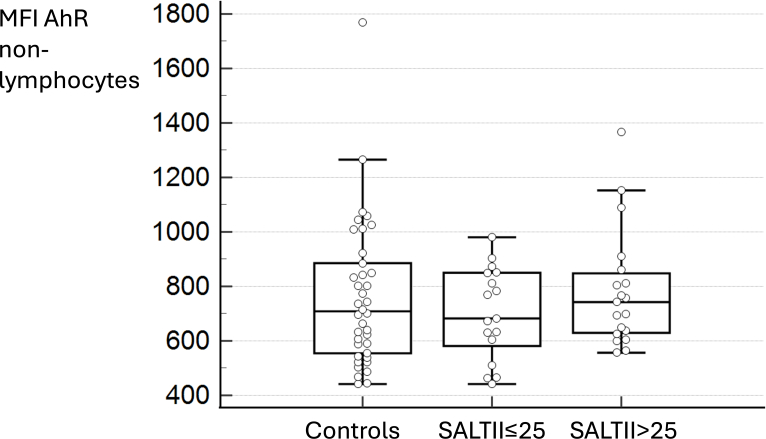


## Data Availability

Data will be made available on request.
